# [^18^F]NaF PET/CT as a Marker for Fibrodysplasia Ossificans Progressiva: From Molecular Mechanisms to Clinical Applications in Bone Disorders

**DOI:** 10.3390/biom14101276

**Published:** 2024-10-10

**Authors:** Jolien Zwama, Neeltje M. Rosenberg, Vincent A. Verheij, Pieter G. H. M. Raijmakers, Maqsood Yaqub, Esmée Botman, Ruben D. de Ruiter, Mark R. Garrelfs, Arend Bökenkamp, Dimitra Micha, Lothar A. Schwarte, Bernd P. Teunissen, Adriaan A. Lammertsma, Ronald Boellaard, Elisabeth M. W. Eekhoff

**Affiliations:** 1 Amsterdam UMC location Vrije Universiteit Amsterdam, Department of Endocrinology and Metabolism, De Boelelaan 1117, Amsterdam, The Netherlands; 2 Amsterdam Movement Sciences, Tissue Function and Regeneration, Amsterdam, The Netherlands; 3 Amsterdam Reproduction and Development, Amsterdam, The Netherlands; 4 Rare Bone Disease Centre, Amsterdam, The Netherlands; 5 Amsterdam UMC location Vrije Universiteit Amsterdam, Department of Radiology and Nuclear Medicine, De Boelelaan 1117, Amsterdam, The Netherlands; 6 Dijklander Hospital, Maelsonstraat 3, 1624 NP Hoorn, The Netherlands; 7 Amsterdam UMC location University of Amsterdam, Department of Pediatric Endocrinology, Emma Children’s Hospital, Meibergdreef 9, Amsterdam, The Netherlands; 8 Amsterdam UMC location University of Amsterdam, Department of Pediatric Nephrology, Emma Children’s Hospital, Meibergdreef 9, Amsterdam, The Netherlands; 9 Amsterdam UMC location Vrije Universiteit Amsterdam, Department of Human Genetics, De Boelelaan 1117, Amsterdam, The Netherlands; 10 Amsterdam UMC location Vrije Universiteit Amsterdam, Department of Anesthesiology, De Boelelaan 1117, Amsterdam, The Netherlands; 11 Department of Nuclear Medicine and Molecular Imaging, University of Groningen, University Medical Center Groningen, Groningen, The Netherlands

**Keywords:** fibrodysplasia ossificans progressiva, ^18^F-sodium fluoride (^18^F-NaF), heterotopic ossification, musculoskeletal development, osteoblasts, positron emission tomography/computed tomography (PET/CT), rare bone diseases

## Abstract

Fibrodysplasia ossificans progressiva (FOP) is a rare genetic bone disorder characterized by episodic flare-ups in connective tissue, which are frequently followed by the formation of heterotopic ossification. The absence of available plasma-soluble biomarkers for flare-ups or heterotopic bone formation poses severe challenges to the monitoring of disease activity to measure or predict disease progression. Recently, 18-fluor-sodium fluoride positron emission tomography/computed tomography ([^18^F]NaF PET/CT) was introduced as a potential marker for ossifying FOP activity. This review discusses the pharmacokinetics of [^18^F]NaF in relation to the pathophysiology of FOP, and its use as a marker of local bone metabolism in a variety of bone-related disorders. In addition, the review specifically addresses the applicability of [^18^F]NaF PET/CT imaging in FOP as a monitoring modality.

## 1. Introduction

Fibrodysplasia ossificans progressiva (FOP, OMIM #135100) is a devastating and disabling bone disorder caused by a single codon gain-of-function variant in the type 1 bone morphogenetic protein-encoding gene *ACVR1* [1]. The prevalence of FOP is estimated to be approximately one per 1,000,000 [2,3]. Typically, the disease progresses through flare-ups followed by the formation of restricting heterotopic ossifications (HO). A flare-up is an inflammatory-like response which can be induced by trauma, viral infection, or muscle overuse, but it can also occur spontaneously. With some exceptions, flare-ups commonly initiate the formation of HO, which, depending on its localization, causes severe disability through the complete ankyloses of joints. In addition, a chronic component is thought to be present in which HO progresses without apparent flare-ups [4].

Depending on localization, HO can lead to life-threatening complications such as thoracic insufficiency syndrome through ankylosis of the thoracic cage or severe feeding problems due to immobilization of the jaw [5]. HO can ultimately lead to full ankylosis of the entire skeleton. Distinguishing flare-ups with subsequent HO formation from flare-ups that resolve without new bone formation is fundamental to understanding disease progression of this still highly unpredictable condition and essential for measuring treatment responses or weighing the course of action in treatment decisions.

Previous studies on the evaluation of disease activity are yet to identify plasma-soluble biomarkers that reflect disease progression or can effectively predict ossification [6]. C-reactive protein (CRP), alkaline phosphatase (ALP), and several interleukins were researched; some markers seem to be associated with either chronic or acute inflammation, but they are non-specific and do not adequately indicate actual bone formation [7,8]. Current outcome measures are therefore limited to functional mobility assessments, such as the cumulative analogue joint involvement scale (CAJIS) and disease-specific questionnaires such as the FOP Physical Function Questionnaire (FOP-PFQ) [9,10], combined with routine radiology. However, the latter only reveals HO lesions once they are already formed [11]. Magnetic resonance imaging (MRI) could help identify an acute flare-up through evaluation of edema intensity, but image characteristics do not seem to be able to differentiate between ossifying and non-ossifying flare-ups [12].

Fluorine-18 sodium fluoride positron emission tomography combined with computed tomography ([^18^F]NaF PET/CT) is a promising modality for the evaluation of disorders with disturbed bone metabolism [13]. Prior research on FOP demonstrated that ossifying lesions can be identified with [^18^F]NaF PET before HO is evident on CT, along with anatomical information of previously developed HO with combined low-dose CT [13,14]. With this review, we aim to provide an overview of [^18^F]NaF PET applications related to bone disorders, with special focus on the use of [^18^F]NaF PET as a marker for bone metabolism in FOP.

## 2. [^18^F]NaF as a Biological Marker of Bone Formation

### 2.1. Pharmacokinetics of [^18^F]NaF

Based on histological and radiographic data [15,16], the formation of HO in FOP follows a natural pattern of endochondral ossification and bone remodeling. In endochondral ossification, mesenchymal stem cells differentiate into chondrocytes to create a cartilage matrix and turn adjacent mesenchymal stem cells into osteoblasts. The osteoblasts subsequently secrete extracellular organic bone matrix within the cartilage, creating a framework for the mineralization of calcium and phosphate into hydroxyapatite crystals [17,18]. Hydroxyapatite crystals are encapsulated by a hydrated shell, which allows for ionic substitution to alter the physical properties of the crystallinity structure. Fluoride is a natural trace element involved in healthy bone metabolism and one of the ions that can be incorporated in the bone matrix through exchange with hydroxyl groups on the surface of the hydroxyapatite forming fluorapatite [19,20,21]. [^18^F]NaF PET involves the intravenous injection of radioactive [^18^F]NaF, which travels from plasma through the extravascular fluid space, into the hydrated shell from where it becomes incorporated into bone [22]. As [^18^F] decays by positron emission, PET imaging enables the visualization of fluor migration into the crystalline matrix of bone [22]. Gamma-emitting 99m-Technetium (^99m^Tc)-labeled diphosphonates also serves as a radiotracer for regional bone metabolism, but accumulates in areas with increased bone metabolism by chemical adsorption onto the HAP crystal surface and into the crystalline structure. ^99m^Tc has a much longer half-life of six hours compared to the 110 min half-life of ^18^F, and the sensitivity and resolution of PET imaging is significantly better than those of gamma imaging for both bone scans and single-photon emission-computed tomography (SPECT) [23].

**Figure 1:**
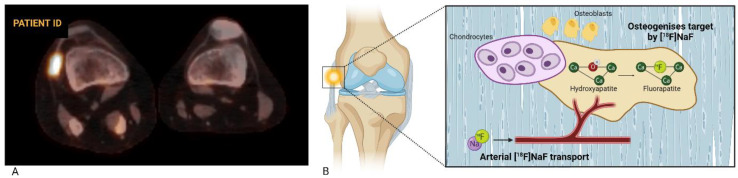
(**A**): Fused axial [^18^F]NaF PET/CT of the right and left knee of a patient with FOP. Tracer uptake appears as orange-to-white, with white indicating a higher metabolic activity. In the right knee, a lesion with increased focal uptake in the lateral collateral ligament is apparent, while no increased uptake is seen in the left knee. (**B**): A schematic overview of [^18^F]NaF uptake incorporation. Chondrocytes create a cartilage matrix, in which adjacent osteoblasts secrete extracellular bone matrix and regulate the mineralization of hydroxyapatite crystals. After intravenous injection, [^18^F]NaF travels through plasma to areas of bone metabolism. Ionic substitution then results in the replacement of an OH-group by [^18^F] in hydroxyapatite forming fluoroapatite.

As recommended by the European Association of Nuclear Medicine (EANM), a static whole-body [^18^F]NaF PET scan can be performed 30–45 min after injection to visualize tracer distribution, but extending the uptake time can improve image quality when assessing the extremities [24]. The acquisition time is dependent on the injected dose (determined by body weight, ranging from 1.5 to 3.7 MBq/kg), uptake time, patient habitus, PET camera, and acquisition parameters, typically ranging from 1 to 5 min per bed position [24,25]. Because uptake of [^18^F]NaF is dependent on hydroxyapatite crystal accessibility and bone blood flow, a [^18^F]NaF PET scan is a marker for local bone metabolism. Consequently, normal bone turnover is also detected by [^18^F]NaF PET, albeit at a significantly lower rate compared to processes with increased bone turnover, such as growth plate development, fracture healing or certain bone diseases [24,26]. Although [^18^F]NaF uptake generally cannot discriminate among different disorders, the spatial pattern of tracer accumulation can be indicative for specific pathophysiological processes [25]. Figure 1A shows a fused [^18^F]NaF PET/CT axial cross section of the right and left knee of a patient with FOP. Increased tracer uptake can be seen in the lateral collateral ligament of the right knee, with no tracer uptake on the contralateral side. Figure 1B shows a schematic overview of the corresponding pharmacokinetics of [^18^F]NaF, demonstrating the pathophysiological process visualized by [^18^F]NaF PET scanning.

Informed consent of the patient was obtained for publication of this scan.

### 2.2. [^18^F]NaF PET in Bone Disorders

In recent years, various studies investigated the use of [^18^F]NaF PET in disorders with abnormal bone metabolism [27]. In several metabolic bone disorders, such as secondary hyperparathyroidism, Paget’s disease and fibrous dysplasia, an association was shown between bone turnover markers known to reflect disease activity, such as ALP and parathormone, and [^18^F]NaF uptake [28,29,30,31,32,33]. More importantly, [^18^F]NaF PET also visualizes abnormal bone metabolism in diseases where bone serum markers do not reflect disease activity, such as melorheostosis [34]. Melorheostosis is a mosaic disorder characterized by hyperostosis of the endosteal and periosteal bone, and sclerosis of the spongious bone, associated with increased bone remodeling and abnormal mineralization [35]. Jha et al. even demonstrated [^18^F]NaF uptake in lesions not yet evident on CT, suggesting that [^18^F]NaF PET is an early marker of disease activity [36].

In inflammatory bone disorders, such as rheumatoid arthritis and ankylosing spondylitis, [^18^F]NaF PET was able to identify sites with active bone formation rather than inflammation [37,38]. This was confirmed by a study by Bruijnen et al. in which bone biopsies of [^18^F]NaF PET-positive lesions revealed active bone formation [39]. In rheumatoid arthritis, significantly higher uptake values were found in erosive joints compared to non-erosive joints, and accumulation of [^18^F]NaF was associated with ongoing bone damage [40]. [^18^F]-Fluorodeoxyglucose (FDG) PET also showed increased tracer uptake in erosive joints, where it concentrated in the joint space, whereas [^18^F]NaF uptake was measured in the bone cortex, likely reflecting upregulated bone turnover.

There is a wide variety of indications beyond bone disorders for which a [^18^F]NaF PET is applicable, such as for the assessment of arterial mineral deposition and bone malignancies [24]. In orthopedics, [^18^F]NaF PET/CT is used for evaluating potential loosening of prostheses [41,42] and bone regeneration after placement of allogeneic bone graft [43]. In addition, [^18^F]NaF PET identified the presence of HO formation in the right gluteal region after surgical internal fixation of the right hip, demonstrating its potential as a marker for HO formation in FOP [44].

These studies provide evidence for the potential of [^18^F]NaF PET to visualize local bone metabolism in a variety of bone disorders. Both the ability to detect new bone formation in lesions not yet evident on CT and the evaluation of bone turnover rather than inflammation, make it a potential tool for the assessment of HO in FOP.

## 3. [^18^F]NaF PET/CT in FOP

Before [^18^F]NaF PET/CT was introduced as marker for FOP, Kaplan et al. used ^99m^Tc-labeled diphosphonates bone scans to show natural bone remodeling of HO in patients with FOP. They showed increased tracer uptake in newly developing lesions and a comparable uptake rate of mature lesions to the normotopic skeleton [16]. However, a more recent study showed that bone scans failed to identify active bone formation in the early stage of disease, as out of all 41 FOP patients with clinical flare-up symptoms within one year prior to scanning, no abnormal tracer uptake was detected, while almost all flare-ups led to HO formation [45]. Consequently, bone scans do not seem to provide additional value beyond routine radiology. Currently, whole-body CT scanning is the primary imaging modality to monitor disease progression. Even in a low-dose setting, CT provides accurate 3D-rendering of heterotopic ossification [46] and is readily available in most hospitals. Yet, its functionality may be limited, as it only shows mature HO, which is the end stage of an ossifying flare-up cascade. CT lacks the ability to show the active process of bone formation and thus fails to detect lesions in an earlier stage, when endochondral ossification started but no clear depictable heterotopic bone formed yet [16]. Molecular or nuclear imaging, in particular [^18^F]NaF PET/CT, on the other hand, has the capacity to visualize both mature HO and localizations of active ossification. The increased osteoblast activity during HO formation results in [^18^F] accumulation in active disease sites. The addition of [^18^F]NaF PET as a dedicated marker for bone metabolism facilitates a more panoramic detection of lesions, and more importantly, allows to distinguish between ossifying flare-ups and non-ossifying flare-ups [12,13,14].

At present, [^18^F]NaF PET/CT was used in nine studies as a marker for FOP, see Table 1. The objectives for the use of [^18^F]NaF PET/CT can broadly be divided into two categories, namely for the early detection of ossification and the evaluation of disease activity over time.

### 3.1. Early Detection of Ossification

**Figure 2:**
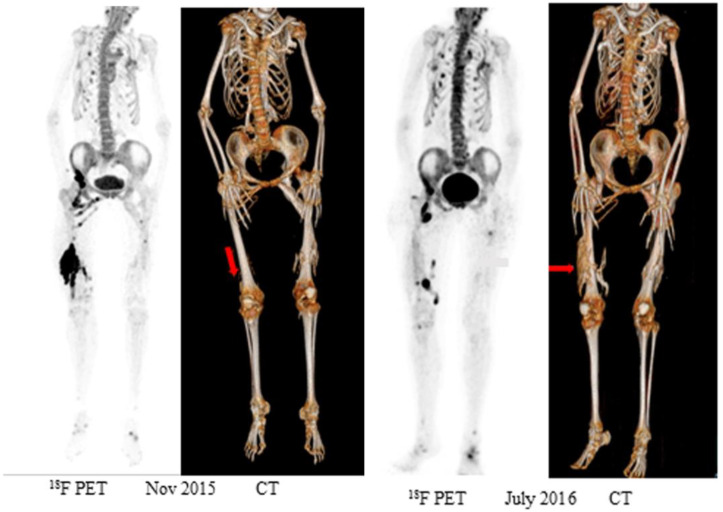
Whole-body [^18^F]NaF PET maximum intensity projection (MIP) (left panel) and corresponding low-dose CT 3D reconstruction (right panel) of a patient with FOP (Nov. 2015) and consecutive scan (July 2016). [^18^F]NaF tracer uptake is displayed on a grey scale, with black indicating a higher tracer accumulation. [^18^F]NaF PET shows initially a lesion with increased tracer uptake in the upper right leg in the quadriceps muscle, while the corresponding CT shows no apparent HO. Eight months later, the increased tracer uptake in the right quadriceps muscle subsided, while the low-dose CT shows a large HO lesion, analogous to the site of increased tracer uptake in Nov. 2015. (Reprinted from Bone, Vol. 109, Eekhoff EMW, Botman E, Coen Netelenbos J, et al., [^18^F]NaF PET/CT scan as an early marker of heterotopic ossification in fibrodysplasia ossificans progressiva, 143–146, Copyright (2018), with permission from Elsevier.).

Eekhoff et al. first described how postoperative ossification could be detected with [^18^F]NaF PET just one month after maxillofacial surgery in a FOP patient with a completely restricted mouth opening, while new HO was not yet evident on CT [13]. The regions with increased tracer uptake illustrated that [^18^F]NaF was a marker for the formation of HO, as the patient’s jaw quickly re-ankylosed from ossification that was visualized on a follow-up CT. Similar findings were later reported in FOP mouse models demonstrating increased tracer uptake during the formation of HO, which subsequently reduced during bone maturation coinciding with reducing osteoblast activity [47]. Consequently, Eekhoff et al. performed a [^18^F]NaF PET/CT on a 19 y/o girl just 3 weeks after she presented with a spontaneous swelling of her right upper leg [14]. While the swelling spanned her entire right upper leg, the [^18^F]NaF PET/CT showed high [^18^F]NaF uptake only at circumscriptive locations in the distal quadriceps muscle, without evidence of HO on concurrent CT (Figure 2). Interestingly, when the flare-up disappeared 8 months later, a follow-up [^18^F]NaF PET/CT scan showed markedly decreased muscle [^18^F]NaF uptake in this location, while maturing HO was visible exclusively at the location where the [^18^F]NaF PET/CT scan was active initially. This principle was endorsed by the study of Botman et al., who demonstrated that flare-ups with severe edema on MRI did not result in new HO if there was no significant [^18^F]NaF uptake [12].

### 3.2. Chronic Lesions and Clinical Trials

Botman et al. showed another distinct purpose of the [^18^F]NaF PET/CT scan, namely the ability to monitor activity of chronic lesions in FOP patients, which often progress without typical symptoms (11). In four out of the five FOP patients studied, one or more asymptomatic HO lesions showed increased [^18^F]NaF uptake with volumetric progression on subsequent scans, confirming the existence of an asymptomatic chronic course in FOP that is not directly related to the presence of a clinically apparent flare-up.

In addition to early detection of ossifying lesions and detection of asymptomatic chronic lesions, PET imaging allows for quantification of tracer uptake as a potential measure of disease activity that can be used to follow-up lesions sequentially and in the setting of clinical trials. For this reason, interest in this modality in clinical research for FOP grew, and full-body [^18^F]NaF PET/CT was incorporated in two recent Phase 2 trial protocols investigating novel therapies aiming to reduce HO formation [51,53]. While no data are yet available from the ongoing STOPFOP trial, the LUMINA-1 trial used [^18^F]NaF PET/CT to measure osteogenic activity of pre-existing lesions and new HO lesions serially, and to differentiate mineralizing lesions from mature HO [51].

## 4. Quantitative [^18^F]NaF PET

The gold standard for quantifying tracer uptake is a demanding procedure, which requires a dynamic scan protocol, arterial sampling, and tracer kinetic modeling [54]. A 60 min PET scan starts with [^18^F]NaF tracer injection, followed by collection of arterial blood samples. Using PET pharmacokinetic models and nonlinear regression analysis (NLR) to fit the observed time activity curves, the underlying molecular targets and processes can be quantified, making it possible to estimate the net rate of [^18^F]NaF incorporation on the crystal surface (*K_i_*). The net influx rate accounts for inter- and intra-patient variations in the input function, i.e., the activity of [^18^F]NaF in arterial blood over time, representing the availability of the tracer from blood to tissue. Moreover, it is a more representative pharmacokinetic parameter for ^18^F trapping in bone. Previous studies demonstrated a significant correlation between *K_i_* and bone histomorphometric parameters indicative of bone formation [31,33,55]. Even though this method is most accurate for quantifying tracer uptake, the complexity and long scan duration hinders its use in clinical practice, necessitating the use of static scans with simplified parameters, such as standardized uptake value (SUV), to quantify tracer uptake. A static scan captures the tracer distribution at a specific uptake time interval, typically after 30–45 min post-injection. It involves a much shorter scan and does not require arterial sampling [24]. This is particularly relevant for patients with FOP, who may find it difficult to lie for 60 min and in whom blood withdrawal is not without risk and can initiate flare-ups with subsequent bone formation [56].

SUV is a dimensionless measure that represents tracer activity concentration in a delineated volume of interest (VOI), corrected for the total injected activity and tracer distribution volume (e.g., body weight or lean body mass) [57]. The most frequently used SUV metrics are SUV_mean_ (mean tracer uptake in a VOI), SUV_max_ (tracer uptake in the voxel in a VOI that has the highest uptake) and SUV_peak_ (mean uptake in a 1 cm^3^ sphere having the highest average value across all positions within the lesion), respectively. Even though SUV_max_ is one of the most widely used simplified parameters, it is susceptible to technical factors, such as image noise, as it merely represents a single voxel value [58]. Although SUV is a static measurement at a single time point, the uptake of tracer from plasma to bone is a dynamic process that is affected by biological factors such as the tracer concentration in blood and tracer transport. It should be noted that SUV does not take into account these factors, and is therefore not able to differentiate specific uptake onto the bone crystal surface from background activity, or account for biological changes in delivery due to, for example, therapy. Nevertheless, research shows a significant correlation between SUV_mean_ and NLR-derived *K_i_* in healthy volunteers and patients undergoing bone surgery, as well as a significant correlation between change in SUV_mean_ and NLR-derived *K_i_* [57]. Furthermore, a significant correlation was found between [^18^F]NaF SUV and a bone histomorphometric parameter indicative of bone formation in patients with multiple myeloma [55]. These findings suggest that SUV could serve as a viable alternative to the gold standard dynamic analysis, particularly in routine clinical practice. Although, when using SUV to measure treatment response, it is essential to validate changes in SUV with the gold standard dynamic analysis to determine whether treatment itself has an effect on biological factors influencing tracer uptake.

Six out of eight studies involving [^18^F]NaF PET in FOP patients reported the use of quantitative measurements to assess disease activity, as shown in Table 2. One [^18^F]NaF PET study compared NLR-derived K_i_ with simplified uptake parameters to assess their potential when evaluating the formation of HO in FOP patients [53]. In PET-active lesions, the correlation between mean target to blood ratio (TBR_mean_) and NLR-derived *K_i_* was stronger than the correlation between SUV_mean_ and NLR-derived *K_i_*. TBR_mean_ is calculated by the dividing the SUV_mean_ of a lesion by the image-derived SUV_mean_ in whole blood, accounting for possible variations in blood pool activity over time, which can be obtained from static imaging as well and may be a better alternative to monitor response than changes in uptake derived from SUV metrics.

Two different studies reported different SUV-based thresholds to identify active HO lesions. Botman et al. used [^18^F]NaF PET/CT in five patients to follow up on HO over time [48]. By measuring the average SUV_peak_ in the left and right supra-acetabular region, a threshold value of 8.4 was established for active HO. Volumetric progression of HO was observed in lesions with tracer uptake above this threshold value, even in the absence of clinical symptoms of a flare-up, while HO without increased tracer uptake demonstrated no volumetric progression. In the LUMINA-1 randomized controlled trial, a patient-specific threshold of pathologic tracer uptake was defined by SUV_max_ [51]. A lesion was considered active when tracer uptake would exceed three times the normal SUV_mean_ in the supra-acetabular region.

## 5. Discussion

In recent years, [^18^F]NaF PET/CT became an increasingly used tool for the evaluation of bone metabolism in FOP, where it served diverse purposes, such as evaluating flare-ups post-intervention and monitoring the natural progression of disease. The findings demonstrate an association between increased tracer uptake and subsequent HO formation, consistent with observations in various bone disorders where [^18^F]NaF uptake seems to correlate with bone serum markers and histomorphology. Given the absence of markers for disease activity in FOP, and the unpredictable clinical manifestation during disease progression, [^18^F]NaF PET/CT is currently the only tool to measure or predict active HO formation.

Disease progression in FOP seems to follow two distinct patterns, i.e., HO formation accompanied by acute flare-ups and HO expansion in the absence of clinical signs. Botman et al. demonstrated that in both processes, [^18^F]NaF accumulated and established a cut-off value for increased uptake in HO measured as SUV_peak_ > 8.4 to be predictive for HO expansion or formation [48]. However, it should be noted that the time of scanning relative to lesion development has great impact on uptake values. For instance, in a follow-up case report on limb surgery, a [^18^F]NaF PET/CT conducted fourteen days post-intervention showed a SUV_max_ of only 6.4 at the surgical site, suggesting no disease progression, yet new HO was confirmed by CT after eight weeks [50]. Longitudinal [^18^F]NaF PET studies on the natural progression of disease are needed to determine the time course of tracer accumulation and the ideal time point of scanning during a flare-up. Studies with a larger cohort should consider using receiver operating characteristic (ROC) analysis to determine the optimal threshold value predictive for HO formation.

All but one study on [^18^F]NaF PET/CT in patients with FOP used a static scan and simplified uptake parameters to reflect disease activity. Various SUV variations are used to measure tracer uptake, but so far only one study validated the use of simplified parameters against full dynamic analysis in FOP patients [52]. In a report by de Ruiter et al., dynamic analysis was performed in seven patients at baseline and follow-up as part of the LUMINA-1 randomized controlled trial with garetosmab. This study suggests that when monitoring PET-active lesions over time, TBR should be considered as an appropriate alternative to full kinetic analysis. However, more research is required on the appropriate cut-off value when using TBR, particularly because various cut-off values of different simplified parameters were proposed [48,51].

Currently, there is no worldwide-approved treatment to prevent or stop HO formation in FOP. Guidelines on medical management provided by the International Clinical Council on FOP recommend corticosteroid treatment in case of a flare-up when it involves a major joint, the jaw, or submandibular area, and in situations where HO will likely be formed, such as after major trauma or surgery. Treatment seems most effective when initiated within the first 24 h of a new flare-up. However, the onset of flare-up symptoms can vary greatly between patients, and not all acute flare-ups are followed by HO formation. In addition, a flare-up can persist for months, with symptoms varying significantly between patients, making it challenging to assess whether an ongoing flare-up will initiate HO formation [4]. In such cases, [^18^F]NaF PET has the potential to distinguish ossifying from non-ossifying flare-ups, and could therefore help aim treatment strategies to prevent unnecessary treatment exposure or undertreatment of potential irreversible disease progression.

Despite the potential of [^18^F]NaF PET/CT to be a marker for FOP activity, certain limitations may compromise its use in clinical practice or clinical trials, especially frequent or sequential PET/CT scanning or scanning of pediatric patients. First and foremost, the radiation burden of [^18^F]NaF PET/CT is higher compared to low-dose CT alone and rationale for physicians and medical ethics committees to deter from employing this technique is relatable. However, ongoing advancements in PET detector sensitivity resulted in continuously decreasing necessary effective dosages. Whilst the EANM currently recommends that the administered [^18^F]NaF dose should be between 1.5 and 3.7 MBq/kg in adults (22), it was shown that a dose of just 1.2 MBq/kg preserves the diagnostic value in more advanced PET/CT scanners in patients with FOP (13). To contextualize radiation dosimetry; an 80 kg adult receiving 3.7 MBq/kg results in an effective dose of approximately 7.1 mSv from a [^18^F]NaF PET, whereas a 1.2 MBq/kg dose results in a radiation dose of around 2.4 mSv. Additionally, although the radiation exposure associated with the CT component can be as high as 8 mSv for a diagnostic CT, an average low-dose CT contributes about 2.5 mSv to a combined [^18^F]NaF PET/CT, resulting in a total effective radiation dose of roughly 3.5–5.0 mSv [14,24]. Nonetheless, the advantages of [^18^F]NaF PET/CT must always be balanced against the increased radiation exposure to the patient.

Secondly, compared to CT imaging, which typically only takes two minutes, PET imaging requires a significantly longer acquisition time, extending up to 30 min for a static scan and 60 min for a dynamic scan. These long scan times might cause discomfort in patients with FOP because of painful HO and increased risk of skin and nerve damage due to pressure points. It is therefore important to ensure a comfortable position by using special care mattresses or pillows and to reserve ample time to put the patient on the scan bed.

Furthermore, due to the relatively high costs compared to conventional imaging modalities, and the logistically complex tracer production, [^18^F]NaF PET imaging might not be available in all centers seeing FOP patients. Additionally, local expertise is essential for the successful execution and interpretation of [^18^F]NaF PET scans. When PET scanners or the [^18^F]NaF tracer are not available, physicians can resort to a ^99m^Tc-labeled diphosphonates SPECT/CT scan as a more conventional alternative to [^18^F]NaF PET/CT to visualize regional bone metabolism. However, this could reduce diagnostic accuracy, as comparative studies demonstrated that [^18^F]NaF PET/CT is more precise in detecting both malignant and benign lesions and has better quantitative properties compared to SPECT/CT [23].

A new breakthrough in PET imaging is the development of long axial field-of-view (LAFOV) scanners, which have the potential to mitigate some of these limitations. Due to the limited axial field-of-view of conventional PET scanners, multiple bed positions are required for a whole-body PET scan, leading to an extended scan duration. The axial field of view of whole-body LAFOV scanners typically exceeds 1 m, which, in combination with increased detector sensitivity, leads to an impressive reduction in the total scan duration to two minutes without compromising image quality [59]. In children, the reduction in scan time may also limit the need for anesthesia to reduce motion artifacts. In addition, the increased detector sensitivity allows for a reduction in tracer dose, making [^18^F]NaF PET a more attractive modality for pediatric patients or for follow-up image assessment [59]. These features also enable the possibility for a shortened non-invasive whole-body dynamic scan protocol, as was recently demonstrated by van Sluis, et al. with ^18^F-FDG in cancer research [60]. By eliminating the need for blood sampling and reducing the total scan duration, the initial constraints of gold standard quantification are significantly reduced, leading to the possibility of a patient-friendly whole-body dynamic scan protocol. While these recent advancements certainly seem promising, further research is required to validate its feasibility in [^18^F]NaF PET or FOP.

## 6. Conclusions

At present, [^18^F]NaF PET/CT is the only available biological marker for HO formation in FOP. This review provides an overview of [^18^F]NaF PET/CT use since its introduction for FOP in 2016. Due to the capacity to localize and quantify regional bone metabolism, [^18^F]NaF PET/CT is able to measure active disease sites during acute flare-ups as well as in asymptotic lesions. It has the potential to differentiate between ossifying and non-ossifying flare-ups, making it a potential valuable tool in the clinical evaluation of flare-ups. [^18^F]NaF PET/CT imaging allows for precise assessment of regional bone metabolism over time, enhancing the understanding of the natural progression of disease, and enabling new outcome measures for clinical trials. However, additional research is required to further specify the relation between tracer uptake and disease activity, and to establish a standardized approach for [^18^F]NaF PET/CT interpretation.

## Figures and Tables

**Table 1:** Studies using [^18^F]NaF PET/CT for the evaluation of FOP.

Ref.	Authors	Study type	Objective of [^18^F]NaF PET/CT use
[13]	Eekhoff et al. (2017)	Follow-up case study	To evaluate possible flare-ups after surgery
[47]	Upadhyay et al. (2017)	Follow-up animal study	To evaluate the mineralization of HO
[14]	Eekhoff et al. (2018)	Case study	To evaluate a flare-up over time
[48]	Botman et al. (2019)	Follow-up study	To monitor the natural progression of disease
[49]	Botman et al. (2020)	Case study	To evaluate a possible flare-up after radiotherapy
[12]	Botman et al. (2020)	Case study	To assess the diagnostic value of MRI compared to [^18^F]NaF PET
[50]	Botman et al. (2020)	Case study	To evaluate a possible flare-up after surgery
[51]	Di Rocco et al. (2023)	Randomized controlled trial	To measure treatment response (LUMINA-1)
[52]	De Ruiter et al. (2024)	Substudy LUMINA-1	To assess the use of simplified uptake parameters compared to full kinetic analysis

HO = heterotopic ossification, MRI = magnetic resonance imaging.

**Table 2:** Quantitative assessment of studies using [^18^F]NaF PET/CT for the evaluation of FOP patients.

Ref.	Authors	Study Type (n)	Quantitative [^18^F]NaF PET Parameters	Quantitative [^18^F]NaF PET and Follow-Up CT Results
[13]	Eekhoff et al. (2017)	Case study (1)	SUV_mean_	SUV_mean_ measured in masseter muscle of 12.4, 23.3, 10.6 (left), and 19.0, 16.2, 9.6 (right), one month, six months and twelve months post-surgery, respectively. Both sites proceeded with HO formation.
[14]	Eekhoff et al. (2018)	Case study (1)	No quantitative assessment reported	-
[48]	Botman et al. (2019)	Retrospective follow-up study (5)	SUV_peak_	Average SUV_peak_ of normotopic bone at the supra-acetabular region in five patients was measured at 5.5 (SD 1.4). A cutoff value of SUV_peak_ > 8.4 was established for PET active lesions indicating volumetric progression of HO.
[49]	Botman et al. (2020)	Case study (1)	No quantitative assessment reported	-
[12]	Botman et al. (2020)	Retrospective follow-up study (4)	SUV_peak_	Flare-ups visualized by MRI without pathologically increased [^18^F]NaF uptake (SUV_peak_ > 8.4) did not result in HO progression.
[50]	Botman et al. (2020)	Case study (1)	SUV_max_	SUV_max_ of 6.4 measured in distal femur fourteen days post through the knee amputation. Follow-up CT eight weeks post-surgery confirmed new HO formation.
[51]	Di Rocco et al. (2023)	Randomized controlled trial LUMINA-1	SUV_max_, SUV_mean_, SUV_peak_, TLA	A patient-specific threshold value was used to identify PET active lesions. Normal uptake was measured in the supra-acetabular region using SUV_mean_. A lesion was considered active when SUV_max_ exceeded three times the normal uptake value. In addition to SUV_max_, SUV_mean_, SUV_peak_, and TLA were analyzed over time.
[52]	De Ruiter et al. (2024)	Substudy LUMINA-1 (7)	NLR derived *K_i_*, SUV_mean_, TBR_mean_	Static SUV_mean_ correlated with NLR-derived *K_i_* at baseline and one year follow-up. Static TBR_mean_ correlated with NLR-derived *K_i_* at baseline and one year follow-up. Change in static SUV_mean_ and TBR_mean_ measured in HO did not correlate with change in NLR-derived *K_i_*. Change in static SUV_mean_ and TBR_mean_ measured in PET active lesions (SUV_peak_ > 8.4) correlated with change in NLR-derived *K_i_*.

HO = heterotopic ossification, MRI = magnetic resonance imaging, SD = standard deviation, SUV = standardized uptake volume, TBR = target to blood ratio, and TLA = total lesion activity (product of the sum of lesion SUV_mean_ multiplied by metabolic volume).

## Data Availability

No new data were created or analyzed in this study. Data sharing is not applicable to this article.
